# A Marine Collagen-Based 3D Scaffold for In Vitro Modeling of Human Prostate Cancer Niche and Anti-Cancer Therapeutic Discovery

**DOI:** 10.3390/md22070295

**Published:** 2024-06-26

**Authors:** Won Hoon Song, Ye Seon Lim, Ji-Eun Kim, Hae Yeong Kang, Changyong Lee, Lata Rajbongshi, Seon Yeong Hwang, Sae-Ock Oh, Byoung Soo Kim, Dongjun Lee, Yong Jung Song, Sik Yoon

**Affiliations:** 1 Department of Urology, Pusan National University Yangsan Hospital and Pusan National University College of Medicine, Yangsan 626-870, Republic of Korea; luchen99@hanmail.net; 2 Department of Anatomy and Convergence Medical Sciences, Pusan National University College of Medicine, Yangsan 626-870, Republic of Korea; yeseonlim@pusan.ac.kr (Y.S.L.); osldy@naver.com (J.-E.K.); kang-hy1703@hanmail.net (H.Y.K.); qhrrn79@naver.com (C.L.); latapharm@gmail.com (L.R.); anatomy2017@pusan.ac.kr (S.Y.H.); wldmsdl9611@gmail.com (S.-O.O.); 3 Immune Reconstitution Research Center of Medical Research Institute, Pusan National University College of Medicine, Yangsan 626-870, Republic of Korea; gynsong@gmail.com; 4 School of Biomedical Convergence Engineering, Pusan National University, Yangsan 50612, Republic of Korea; bskim7@pusan.ac.kr; 5 Department of Convergence Medicine, Pusan National University College of Medicine, Yangsan 50612, Republic of Korea; lee.dongjun@pusan.ac.kr; 6 Department of Obstetrics and Gynecology, Pusan National University Yangsan Hospital and Pusan National University College of Medicine, Yangsan 626-870, Republic of Korea

**Keywords:** marine collagen, scaffold, hydrogel, cancer stem cell, prostate cancer, 3D cell culture, spheroid, chemoresistance

## Abstract

Recently, the need to develop a robust three-dimensional (3D) cell culture system that serves as a valuable in vitro tumor model has been emphasized. This system should closely mimic the tumor growth behaviors observed in vivo and replicate the key elements and characteristics of human tumors for the effective discovery and development of anti-tumor therapeutics. Therefore, in this study, we developed an effective 3D in vitro model of human prostate cancer (PC) using a marine collagen-based biomimetic 3D scaffold. The model displayed distinctive molecular profiles and cellular properties compared with those of the 2D PC cell culture. This was evidenced by (1) increased cell proliferation, migration, invasion, colony formation, and chemoresistance; (2) upregulated expression of crucial multidrug-resistance- and cancer-stemness-related genes; (3) heightened expression of key molecules associated with malignant progressions, such as epithelial–mesenchymal transition transcription factors, Notch, matrix metalloproteinases, and pluripotency biomarkers; (4) robust enrichment of prostate cancer stem cells (CSCs); and (5) enhanced expression of integrins. These results suggest that our 3D in vitro PC model has the potential to serve as a research platform for studying PC and prostate CSC biology, as well as for screening novel therapies targeting PC and prostate CSCs.

## 1. Introduction 

Prostate cancer (PC) is the second most common cancer and the fifth highest cause of cancer-related mortality among men, with approximately 1.4 million estimated new cases and 375,000 deaths in 2020 [1]. Moreover, PC was the fourth most common cancer across both sexes, posing major clinical challenges and a burden on public healthcare worldwide [1]. Notably, PC is the most common cancer diagnosed among men and the second leading cause of cancer-related mortality among males in the United States, with approximately 288,300 new cases and 34,700 deaths in 2023 [2]. The increasing incidence of PC in recent decades has been driven by factors such as aging, dietary habits, and increased prostate-specific antigen (PSA) testing [3].

Androgen deprivation therapy (ADT) remains the initial treatment for PC owing to the androgen-sensitive nature of PC. However, most patients develop ADT resistance and consequently advance to a more aggressive state known as castration-resistant prostate cancer (CRPC). Current treatment options for advanced PC include the following: chemotherapy with drugs, such as docetaxel and cabazitaxel; new hormone therapies, such as abiraterone and enzalutamide; immunotherapies, such as sipuleucel-T and bispecific T-cell engagers; both external and internal radiotherapy, including radium-223; and targeted therapies [4,5]. Despite substantial advances in the past decade, metastatic CRPC remains highly lethal, with a five-year survival rate of approximately 30% [6]. Given the inadequacy of existing treatments for many patients, the development of new and effective therapies for advanced PC is needed. However, introducing new drugs in the market is challenging. The process from initial discovery to clinical approval is long and costly, typically exceeding 10–15 years and $1–2 billion per drug, with a considerable risk of failure [7]. 

The development of anti-cancer therapeutics relies fundamentally on establishing optimal in vitro tumor models that accurately replicate human tumors, including their drug resistance patterns. This is essential for evaluating the efficacy of potential anti-cancer drugs and understanding their interactions with tumor cells in realistic settings [8]. By simulating the complexities of human tumors in vitro, researchers can predict the effectiveness of new anti-cancer treatments, thereby advancing cancer treatment strategies [9,10]. However, in vitro cell-based assays, which are typically used in preclinical phases, often depend on two-dimensional (2D) cell culture models. In these models, cells grow as monolayers on a flat surface, leading to alterations in the cell morphology, cytoskeletal organization, and intercellular communication [11]. These changes result in modified gene and protein expression patterns that affect various cellular functions, including cell viability, proliferation, behavior, differentiation, and drug sensitivity [8,9,10,11,12]. Consequently, 2D tumor cell cultures fail to fully capture the biological and physiological conditions of actual tumors, including the complexities of their three-dimensional (3D) tissue architecture and functional characteristics related to drug response and resistance, rendering them inadequate for testing and developing anti-cancer therapies. 

Moreover, biosynthesis of drug-metabolizing enzymes, which is crucial for drug toxicity assays, is compromised in monolayer cell cultures [13]. This highlights the need for more advanced and physiologically relevant tumor models to improve the predictive value of preclinical studies in drug development. Notably, over 90% of the drug candidates, almost exclusively screened using 2D assays, failed in clinical trials [14]. Enhancements in drug development methodologies, particularly the transition from 2D to 3D cell culture models, could offer substantial cost-effectiveness, increase drug efficacy, reduce off-target effects, and minimize clinical trial failures [15].

Three-dimensional (3D) cell cultures provide an environment in which cells can grow and interact with the extracellular matrix (ECM) in three dimensions. These models have emerged as valuable tools in cancer research and better mimic the structural and biological characteristics of tumors in vivo [16]. The shift from 2D to 3D models represents a paradigm shift in cancer research, unlocking comprehensive insights into tumor biology and facilitating the development of effective cancer treatments. Common 3D tumor models, such as tumor spheroids and organoids, are particularly noteworthy for their ability to replicate key tumor features, which is essential in preclinical cancer research.

Both histologically and genetically, tumor organoids closely resemble the original tumors from which they are derived. These complex self-organized 3D structures, which are often generated from tissue-resident or pluripotent stem cells, provide revolutionary insights into cancer research. This breakthrough maintains tumor heterogeneity and pathophysiology, including their genetic and functional characteristics [17]. Importantly, organoids naturally form intricate structures that mimic tumor architecture and growth, making them valuable for preclinical drug testing and personalized cancer treatment through patient-derived organoids.

However, despite their remarkable potential, tumor organoids face several substantial hurdles that hinder their widespread application in cancer research. The success rate of organoid establishment varies greatly across different tumors, with high rates in colon cancer and low rates in prostate and pancreatic cancers [18]. Organoid generation and maintenance are a time-consuming and labor-intensive process that requires specific expertise and potentially expensive reagents. The contamination and outgrowth of normal cells and other cell types compromise the purity and functionality of organoid models [18,19], especially in lung cancer [20]. In addition, standardizing and controlling the scalability of organoid production is challenging [21]. Obtaining tumoral tissue often requires invasive procedures, such as biopsies or resections, and organoids generally have a limited lifespan, eventually losing their original characteristics [21]. 

In particular, PC organoids exhibit several notable limitations, such as a low success rate of 15–20%, limited success from primary patient samples, primarily comprising epithelial and stromal cells with fewer tumor cells, and restricted access to clinical specimens, thereby impeding the creation of a diverse organoid bank [22,23,24,25,26].

In contrast, tumor spheroids derived from cancer cell lines or patient samples, which are often cultured using hydrogel-based methods, are pivotal for in vitro models in cancer research. They replicate complex in vivo tumor characteristics, such as multicellular architecture and mass transport barriers, making them superior to 2D models for drug testing and penetration studies [26]. Spheroids are crucial for exploring cell proliferation, metabolism, hypoxia, and tumor heterogeneity in cancer research [27]. Although they are simpler in structure than organoids, which offer a closer genetic and histological match to the original tumors but require complex cultivation, spheroids are widely used in drug screening owing to their accessibility and effectiveness [28]. Despite certain limitations, spheroid models are increasingly recognized as promising tools for drug development and translational oncology research; thus the quest for an optimal 3D in vitro cancer model continues.

Therefore, in this study, we developed an effective 3D in vitro human PC spheroid model using a biomimetic marine collagen-based (MC-B) hydrogel matrix optimized for bioactivity, simplicity, and efficiency. This model incorporates three common PC cell lines (LNCaP, DU-145, and PC3) and demonstrates enhanced malignant properties compared with those of traditional 2D cultures, rendering it suitable for translational oncology research and drug development applications. The characteristics and efficacy of the 3D PC model were assessed. 

## 2. Results

### 2.1. Formation and Growth of PC Cell Spheroids Were Promoted in MC-B Hydrogels 

**Figure 1:**
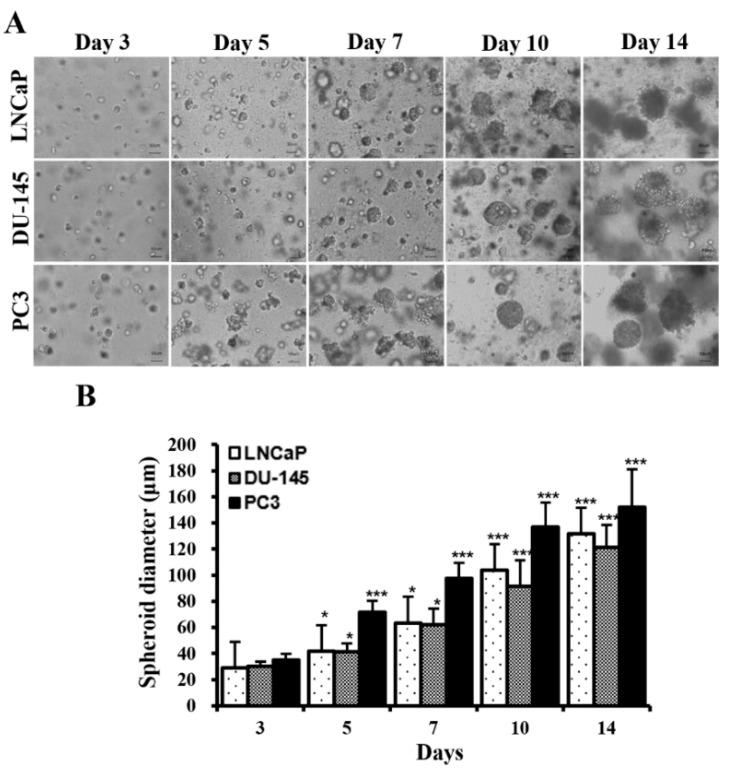
Formation and growth of prostate cancer (PC) cell spheroids in standard plastic tissue culture plates and marine collagen-based hydrogels. (**A**) Phase contrast microscopy images showing PC cell (LNCaP, DU-145, and PC3) spheroids on culture days 3, 5, 7, 10, and 14 (original magnification ×100). (**B**) The diameters of the PC cell spheroids are depicted in a bar graph. Data represent the mean ± standard deviation of three independent experiments. * *p* < 0.05 and *** *p* < 0.001 vs. those on day 1. Scale bars = 50 μm.

Figure 1A shows the phase-contrast microscopy images of 2D and 3D PC cells over time. Three cell types (LNCaP, DU-145, and PC3) began to form multiple spheroids on day 3. The number of spheroids gradually increased with time (Figure 1A). The average diameters of spheroids measured on days 3, 5, 7, 10, and 14 were 29.0, 41.7, 63.4, 103.7, and 131.7 µm, respectively, in LNCaP cells; 30.2, 41.5, 62.2, 91.7, and 121.2 µm, respectively, in DU-145 cells; and 35.0, 71.5, 97.3, 136.7, and 152.2, respectively, in PC3 cells (Figure 1B). Spheroids derived from LNCaP and DU-145 cells were similar in size over time, although the rate of spheroid growth differed slightly depending on the cell type (DU-145 > LNCaP > PC3) (Figure 1A,B). In contrast, PC3 spheroids were significantly larger than LNCaP and DU-145 spheroids after three days of incubation (*p* < 0.001). On day 14 of culture, almost all spheroids from the three cell types were over 120 µm in diameter, suggesting that MC-B hydrogels provide a favorable milieu for the growth of PC cell spheroids, which can be applied in the development of diagnostic and therapeutic strategies for PC.

### 2.2. Proliferation and Clonogenicity of PC Cells Were Enhanced in MC-B Hydrogels 

**Figure 2:**
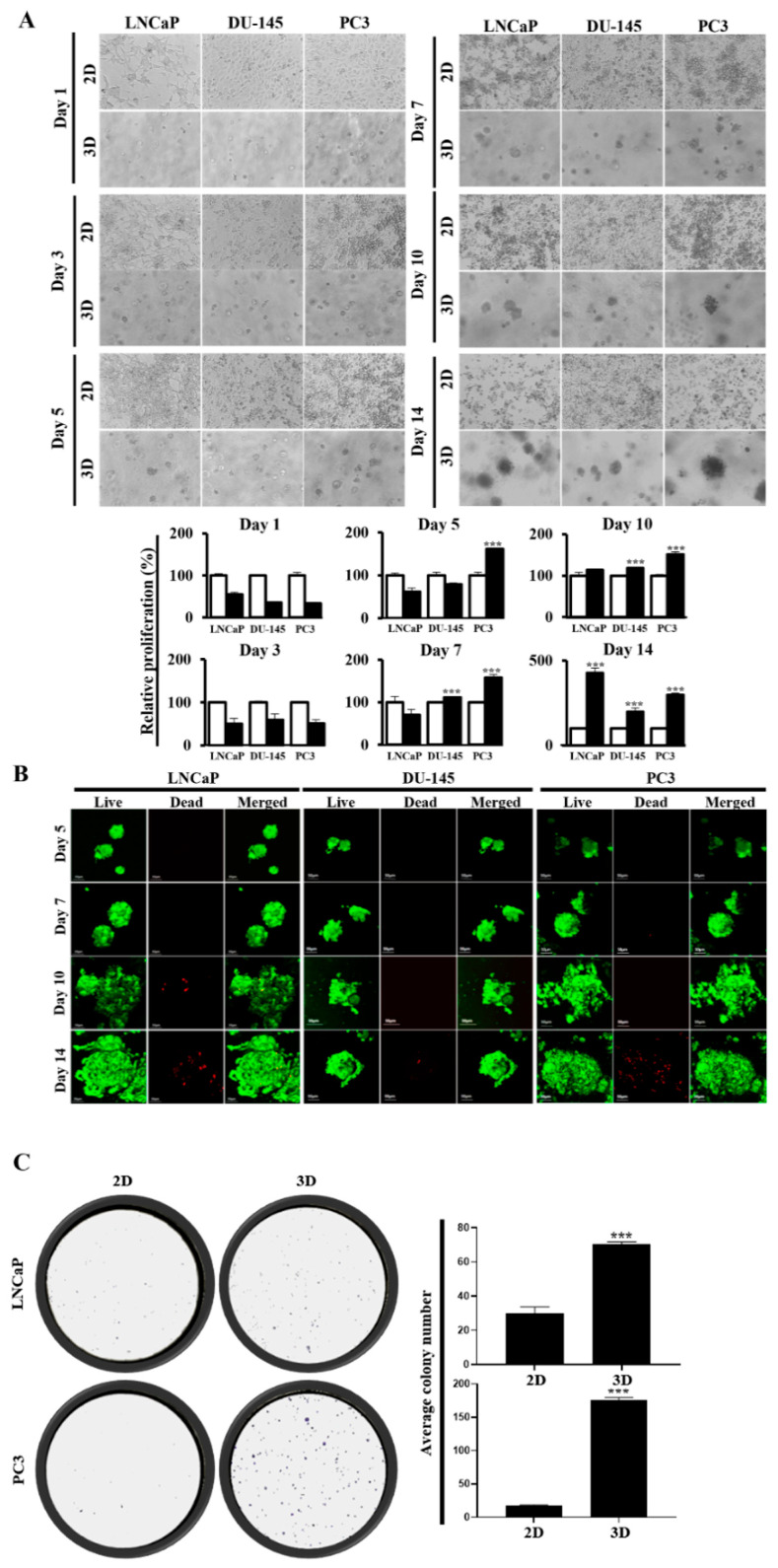
Proliferation of prostate cancer cells in standard plastic tissue culture plates and marine collagen-based hydrogels. (**A**) The water-soluble tetrazolium (WST)-1 assay of LNCaP, DU-145, and PC3 cells in 2D vs. 3D cultures on days 1, 3, 5, 7, 10, and 14. (**B**) Representative fluorescence microscopic image (400×) of 3D-cultured cells on days 5, 7, 10, and 14 as determined using live/dead cell viability assay (Green, calcein: live cells; red, EthD: dead cells). (**C**) The clonogenic assay was used to determine the colony-forming ability of 2D-cultured vs. 3D-cultured LNCaP and PC3 cells over a period of seven days. Data represent the mean ± standard deviation of three independent experiments. *** *p* < 0.001 vs. 2D. Scale bars = 50 μm.

A water-soluble tetrazolium (WST)-1-based colorimetric cell proliferation assay was used to quantify the ability of MC-B hydrogels to facilitate cell proliferation. PC cells were successfully propagated in the MC-B hydrogels. The number of cells in 2D cultures was significantly greater than that in 3D cultures for all three cell types during the first three days of culture (Figure 2A). On day 5, the cell number in 2D cultures was significantly greater than that in 3D cultures of LNCaP and DU-145 cells, whereas PC3 cells showed 16.1-fold higher proliferation in 3D cultures than in 2D cultures (*p* < 0.001) (Figure 2A). On day 7, DU-145 and PC3 cells showed 11.2-fold (*p* < 0.001) and 15.8-fold (*p* < 0.001) higher proliferation, respectively, in 3D cultures than in 2D cultures. After 10 days of culture, the cell numbers in 3D cultures exceeded those in 2D cultures for all three cell types (Figure 2A). On days 10 and 14, the proliferation rates in the 3D and 2D cultures increased 11.4-fold (*p* < 0.01) and 42.6-fold (*p* < 0.001), respectively, for LNCaP cells; 11.9-fold (*p* < 0.001) and 19.8-fold (*p* < 0.001), respectively, for DU-145 cells; and 15.1-fold (*p* < 0.001) and 29.6-fold (*p* < 0.001), respectively, for PC3 cells (Figure 2A). 

Furthermore, these results were consistent with the analysis of cell viability within the spheroids using a live/dead cell assay kit with fluorescence microscopy. Ethidium homodimer-1 staining indicated a compromised cell membrane with subsequent binding to intracellular nucleic acids (red fluorescence), and calcein AM fluorescence indicated metabolically viable cells (green fluorescence). The cells were cultured for 14 consecutive days and assessed on days 7, 10, and 14. Figure 2B shows that almost all cells within 7-, 10-, and 14-day-old spheroids remained viable; however, a few scattered dead cells started to appear on approximately day 10 (Figure 2). Therefore, these results indicate that most PC cells in the MC-B hydrogel remained viable for at least 14 days.

To evaluate the colony-forming ability of the MC-B hydrogels, a clonogenicity assay was performed. On day 7, the 3D MC-B hydrogel culture showed a marked enhancement in colony formation ability by 2.4-fold (*p* < 0.001) in LNCaP cells and 10.4-fold (*p* < 0.001) in PC3 cells compared with those in the 2D culture (Figure 2C). Collectively, these findings indicate that MC-B hydrogels create environmental conditions more suitable for PC cell proliferation and colonization compared to 2D culture. 

### 2.3. Confocal Microscopic Images of PC-Cell-Derived Spheroids 

**Figure 3:**
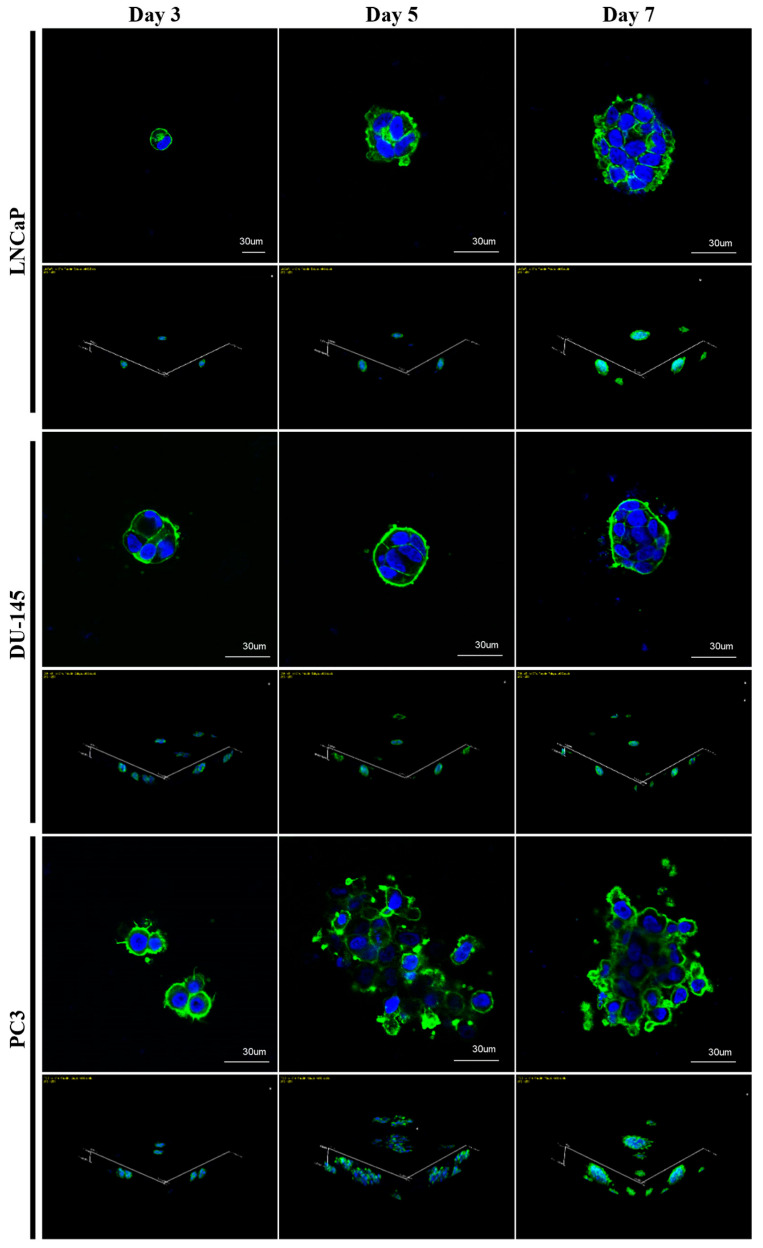
Confocal microscopic images of prostate cancer (PC)-cell-derived spheroids. Morphologies of PC cell lines cultured in marine collagen-based hydrogel. Fluorescence images of F-actin-stained spheroids revealed distinct growth patterns among three cell lines (LNCaP, DU-145, and PC3). Representative optical sections and a Z projection image of F-actin-stained spheroids are shown. Scale bars = 30 μm.

The 3D morphologies of the cell lines were characterized after three, five, and seven days of culture under a confocal microscope. Fluorescence staining of the actin cytoskeleton revealed distinct growth patterns. LNCaP and DU-145 cells spontaneously formed spheroids with a round-type, smooth surface, and compact morphology, as well as with stronger cell–cell contacts, after incubation for three days and became more compact and dense during seven days of culture (Figure 3). These spheroids contained nuclei that were organized regularly around their centers, as assessed by confocal microscopy (Figure 3). 

In contrast, PC3 cells spontaneously started to generate spheroids with a bubble-like appearance after cultivating for three days and became spheroids with irregular surfaces, less compactness, and grape-like morphology after seven days of culture (Figure 3). In addition, PC3 cells were characterized by poor cell-to-cell contact and a disorganized arrangement of nuclei within the spheroids (Figure 3). Notably, PC3 spheroids displayed actin-rich outward protrusions extending into the MC-B hydrogel in all directions. Furthermore, many single cells or clusters of cells were pinched off from the budding areas near the spheroid surface, reminiscent of cancer metastasis (Figure 3). 

### 2.4. Metastatic Potentials of PC Cells Were Elevated in MC-B Hydrogels 

**Figure 4:**
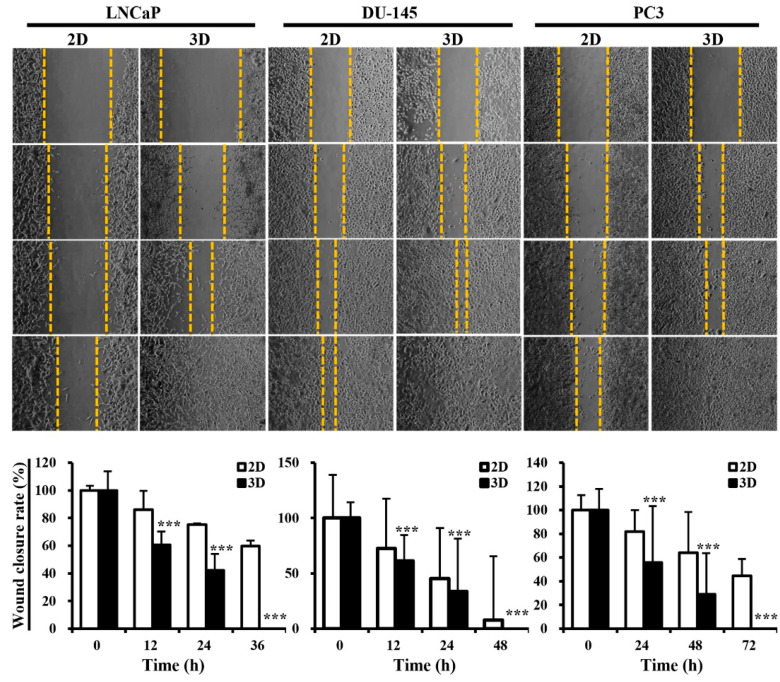
Migratory behavior of prostate cancer cells in standard plastic tissue culture plates and marine collagen-based hydrogels. Representative phase contrast microscopy images from a wound-healing assay of LNCaP, DU-145, and PC3 cells. Images were captured at 0, 12, 24, 36, 48, and 72 h. The distances between the two edges were scaled for three positions at different times. Data represent the mean ± standard deviation of three independent experiments. *** *p* < 0.001 vs. 2D. Scale bars = 50 μm.

Metastasis is a multistep process that includes cancer cell migration and invasion, which are the hallmarks of cancer metastasis. Tumor cells grown in 3D models that can recapitulate the enormous complexity of in vivo biological systems more accurately are considered to exhibit a higher level of metastatic potential than those grown in traditional 2D monolayers. Thus, we hypothesized that 3D multicellular PC spheroids would exhibit enhanced metastatic potential compared to 2D-cultured cells. To assess this, wound healing and invasion assays were performed using LNCaP, DU-145, and PC3 cells. Images of the scratch areas in the wound-healing assay at 0, 12, 24, 36, 48, and 72 h are presented in Figure 4. The rate of wound closure in the 3D cultures was 60.4% (vs. 86.1% for 2D, *p* < 0.001), 42.1% (vs. 75.4% for 2D, *p* < 0.001), and 100% (vs. 59.6% for 2D, *p* < 0.001) at 12, 24, and 36 h, respectively, for LNCaP cells; 61.2% (vs. 72.6 for 2D, *p* < 0.001), 33.6% (vs. 45.2% for 2D, *p* < 0.001), and 100% (vs. 7.8% for 2D, *p* < 0.001) at 12, 24, and 48 h, respectively, for DU-145 cells; and 55.9% (vs. 82.0% for 2D, *p* < 0.001), 29.1% (vs. 64.2% for 2D, *p* < 0.001), and 100% (vs. 44.7% for 2D, *p* < 0.001) at 24, 48, and 72 h, respectively, for PC3 cells (Figure 4). 

**Figure 5:**
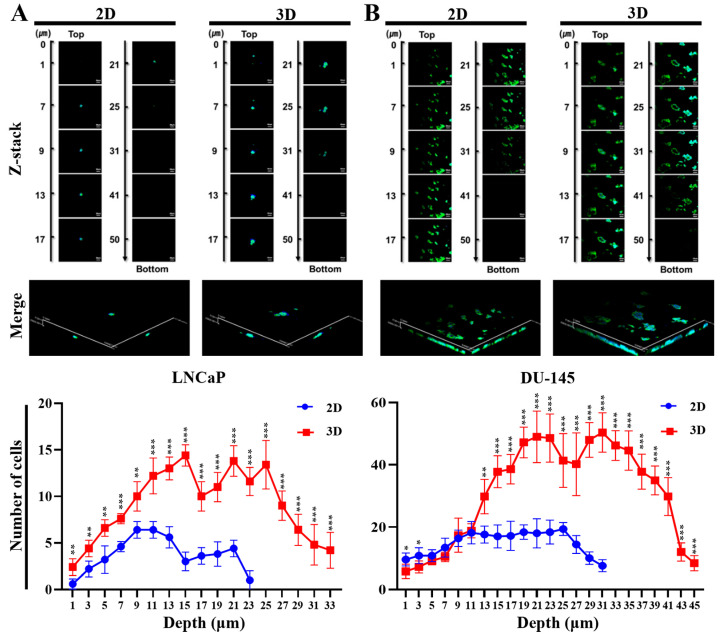
Invasive behavior of prostate cancer cells in standard plastic tissue culture plates and marine collagen-based (MC-B) hydrogels. Maximum intensity projection (x–z) from a z-stack taken with a confocal laser scanning microscope from F-actin stained 2D- and 3D-cultured (**A**) LNCaP and (**B**) DU-145 cells that invaded the 3D MC-B matrix. The 3D culture environment enhanced the invasive ability of LNCaP and DU-145 cells compared to the 2D plate culture environment. Data represent the mean ± standard deviation of three independent experiments. * *p* < 0.05, ** *p* < 0.001, and *** *p* < 0.001 vs. 2D. Scale bars = 50 μm.

Moreover, 3D culture in the MC-B hydrogel significantly increased the number of invading PC cells (red squares) compared to 2D culture (blue circles) across all depths (Figure 5A,B). The 3D culture in the MC-B hydrogel consistently showed significantly higher invasion levels at multiple depths (Figure 5). The maximum invasion depth observed in the 3D cultures was 33 µm (vs. 23 µm for 2D) for LNCaP cells and 45 µm (vs. 31 µm for 2D) for DU-145 cells (Figure 5A,B). Furthermore, in the 3D LNCaP culture, cell invasion peaked at approximately 15 cells at a depth of 25–30 µm, whereas the 2D culture invasion peaked at approximately 7 cells at a similar depth. Peak cell invasion for the 3D DU-145 cell occurred at a depth of approximately 40–45 µm, reaching a maximum value of approximately 55 cells, whereas the 2D culture showed a maximum invasion depth of approximately 20 cells. Collectively, these data provide evidence that the 3D niche provided by the MC-B hydrogels facilitates the migration and invasion of PC cells and promotes their metastatic potential.

### 2.5. Chemoresistance of PC Cells Increased in MC-B Hydrogels 

Multidrug resistance remains a major obstacle to successful cancer chemotherapy. Tumor cells grown in 3D models that can more accurately mimic the properties of living tissues exhibit higher levels of drug resistance than those grown in traditional 2D monolayers. Thus, we hypothesized that 3D multicellular PC spheroids grown within MC-B hydrogels would display enhanced chemotherapeutic resistance to anti-cancer agents for PC compared to 2D-cultured cells. LNCaP and PC3 cells were cultured in the presence of serial concentrations of abiraterone, cisplatin, curcumin, docetaxel, and enzalutamide for 24 and 48 h in both 2D- and 3D-cultured systems. To evaluate the effects of the 3D microenvironment provided by the MC-B hydrogels on drug resistance against various anti-cancer agents in PC cells, cellular cytotoxicity was assessed using a WST-1-based colorimetric cell viability assay. 

**Figure 6:**
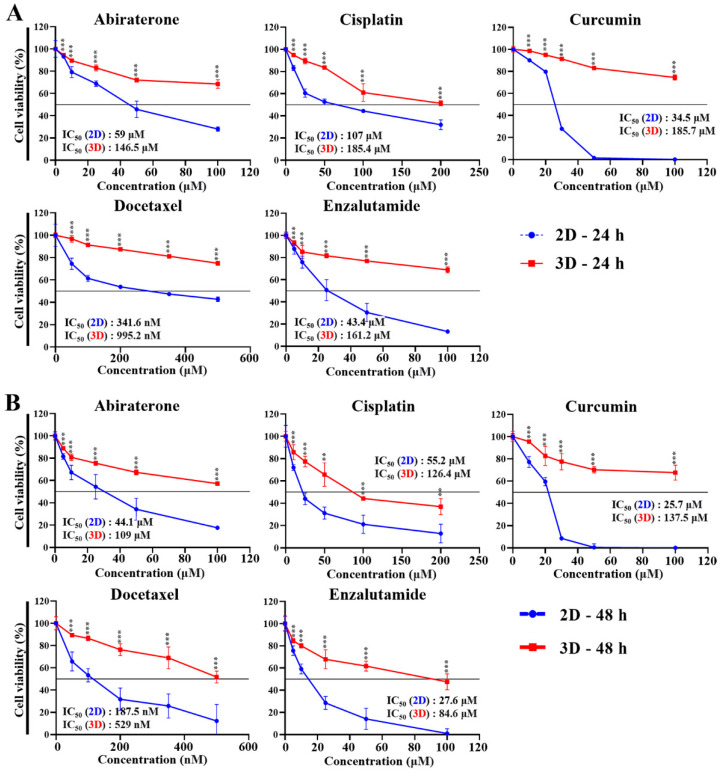

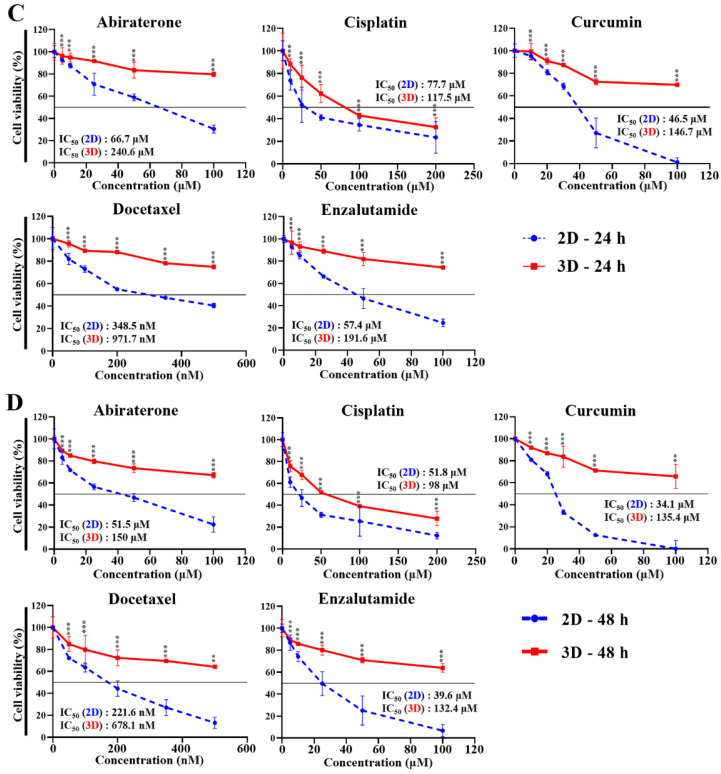
Chemoresistance of prostate cancer cells in standard plastic tissue culture plates and marine collagen-based hydrogels. The cell viability of LNCaP at (**A**) 24 h and (**B**) 48 h, and PC3 at (**C**) 24 h and (**D**) 48 h in 2D and 3D culture after exposure to abiraterone, cisplatin, curcumin, docetaxel, and enzalutamide. Data represent the mean percentage viability ± standard deviation of three independent experiments normalized against untreated control cells. ** *p* < 0.01 and *** *p* < 0.001 vs. 2D.

As shown in Figure 6A, abiraterone treatment for 24 h dose-dependently inhibited the viability of 2D-cultured cells with an IC_50_ value of 59.0 μM, whereas it showed lower responses in 3D-cultured cells (IC_50_ value of 146.5 μM). Similarly, cisplatin and curcumin treatment for 24 h demonstrated reduced effectiveness in 3D cultures compared to 2D cultures, with IC_50_ values of 185.4 and 185.7 μM, respectively, vs. 107.0 and 34.5 μM, respectively, in 2D cultures. Docetaxel and enzalutamide treatment for 24 h also showed a marked difference. For docetaxel, the IC_50_ values were 341.6 and 995.2 nM in 2D and 3D cultures, respectively, and for enzalutamide, the IC_50_ values were 43.4 and 161.2 μM in 2D and 3D cultures, respectively. 

As shown in Figure 6B, dose-dependent abiraterone treatment for 48 h inhibited the viability of 2D-cultured cells with an IC_50_ value of 44.1 μM, whereas it showed lower responses in the 3D culture with an IC_50_ value of 109.0 μM. Similarly, cisplatin and curcumin treatments for 48 h demonstrated reduced effectiveness in 3D cultures compared to 2D cultures, with IC_50_ values of 126.4 and 137.5 μM, respectively, in 3D cultures against 55.2 and 25.7 μM, respectively, in 2D cultures. Treatments with docetaxel and enzalutamide for 48 h also revealed significant differences, with IC_50_ values of 187.5 nM in 2D cultures and 529.0 nM in 3D cultures for docetaxel, and 27.6 μM in 2D cultures vs. 84.6 μM in 3D cultures for enzalutamide.

As shown in Figure 6C, dose-dependent abiraterone treatment for 24 h inhibited the viability of 2D-cultured cells, with an IC_50_ value of 66.7 μM, whereas it showed lower responses in the 3D culture, with an IC_50_ value of 240.6 μM. Similarly, cisplatin and curcumin treatment for 24 h demonstrated reduced effectiveness in 3D cultures compared to 2D cultures, with IC_50_ values of 117.5 and 146.7 μM, respectively, vs. 77.7 and 46.5 μM, respectively, in 2D cultures. Docetaxel and enzalutamide treatment for 24 h also showed a marked difference. For docetaxel, the IC_50_ values were 348.5 and 971.7 nM in 2D and 3D cultures, respectively, and for enzalutamide, the IC_50_ values were 57.4 and 191.6 μM in 2D and 3D cultures, respectively. 

As shown in Figure 6D, dose-dependent abiraterone treatment for 48 h inhibited the viability of 2D-cultured cells with an IC_50_ value of 51.5 μM, whereas it showed lower responses in the 3D system with an IC_50_ value of 150.0 μM. Similarly, cisplatin and curcumin treatments for 48 h demonstrated reduced effectiveness in 3D cultures compared to 2D cultures, with IC_50_ values of 98.0 and 135.4 μM, respectively, against 51.8 and 34.1 μM, respectively, in 2D cultures. Treatments with docetaxel and enzalutamide for 48 h also revealed significant differences, with IC_50_ values of 221.6 nM in 2D cultures and 678.1 nM in 3D cultures for docetaxel, and 39.6 μM in 2D cultures vs. 132.4 μM in 3D cultures for enzalutamide.

### 2.6. Prostate Cancer Stem Cell (CSC) Biomarker Expression Was Augmented in MC-B Hydrogels 

Flow cytometry was used to assess the expression of CSC biomarkers in PC cells cultured under 2D and 3D conditions. The expression of typical prostate CSC surface markers, such as CD44, CD117, and CD133, was evaluated to determine the proportion in human PC cells (LNCaP and PC3) cultured in both 2D and 3D environments.

**Figure 7:**
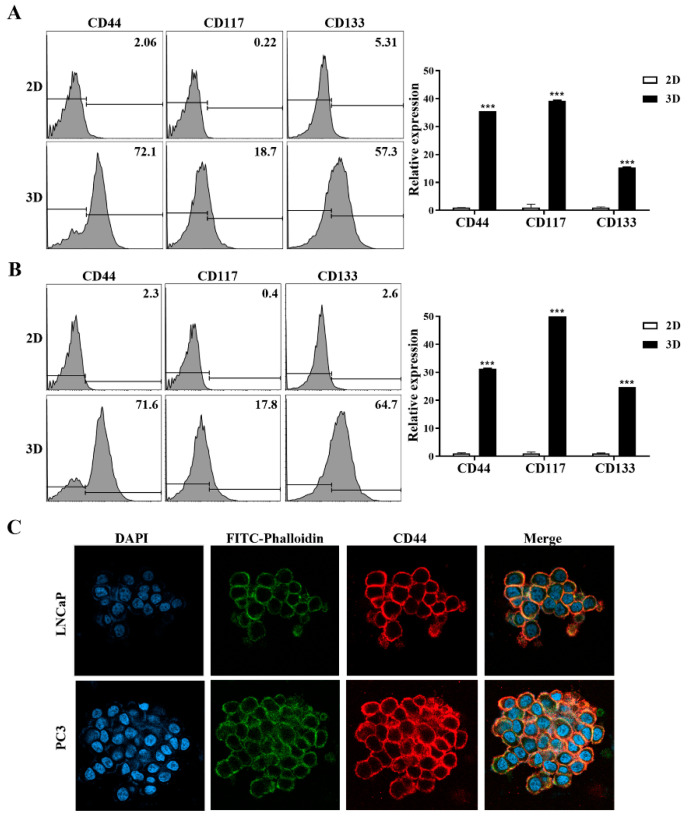
Cancer stem cell surface biomarker expression in standard plastic tissue culture plates and marine collagen-based (MC-B) hydrogels. Flow cytometry displayed a significantly higher CD44, CD117, and CD133 expression in (**A**) LNCaP and (**B**) PC3 cells grown in MC-B hydrogels than in conventional 2D culture on days 7, 10, and 12. Data represent the mean ± standard deviation of three independent experiments. *** *p* < 0.001 vs. 2D. (**C**) Representative image of CD44 cell surface staining visualized using confocal laser scanning microscopy. Each photomicrograph was derived from three independent experiments. Scale bars = 50 μm.

Figure 7A shows the flow cytometry data for LNCaP cells in 2D culture and spheroids on day 7. The percentages of CD44-, CD117-, and CD133-positive LNCaP cells were significantly higher in 3D culture than in 2D culture by 35.6-fold (*p* < 0.001), 39.2-fold (*p* < 0.001), and 15.3-fold (*p* < 0.001), respectively, (Figure 7A). Figure 7B shows the flow cytometry data for PC3 cells in 2D cultures and spheroids on day 7. The percentages of CD44-, CD117-, and CD133-positive PC3 cells were significantly higher in 3D culture than in 2D culture by 31.3-fold (*p* < 0.001), 50.0-fold (*p* < 0.001), and 24.8-fold (*p* < 0.001), respectively (Figure 7B).

CD44 expression levels in LNCaP and PC3 cell lines were determined using immunofluorescence staining, as CD44 is a representative CSC biomarker in PC. After three days of cultivation in MC-B hydrogels, these cells showed intense CD44 staining localized to the plasma membranes (Figure 7C). These results emphasize the value of MC-B hydrogels for the efficient enrichment of CSCs.

### 2.7. Stemness and Pluripotency Marker Expression of PC Cells Were Enhanced in MC-B Hydrogels 

In addition to the flow cytometry and immunocytochemical analyses of prostate CSC biomarker expression, the expression of key genes associated with cancer stemness and pluripotency was evaluated to determine the CSC enrichment efficiency of our 3D PC cell culture model. Based on the results of flow cytometric analysis, we isolated CD117^−^ and CD117^+^ cells from the 3D-cultured MC-B hydrogel cells using fluorescence-activated cell sorting (FACS) for further analysis of their molecular signatures.

**Figure 8:**
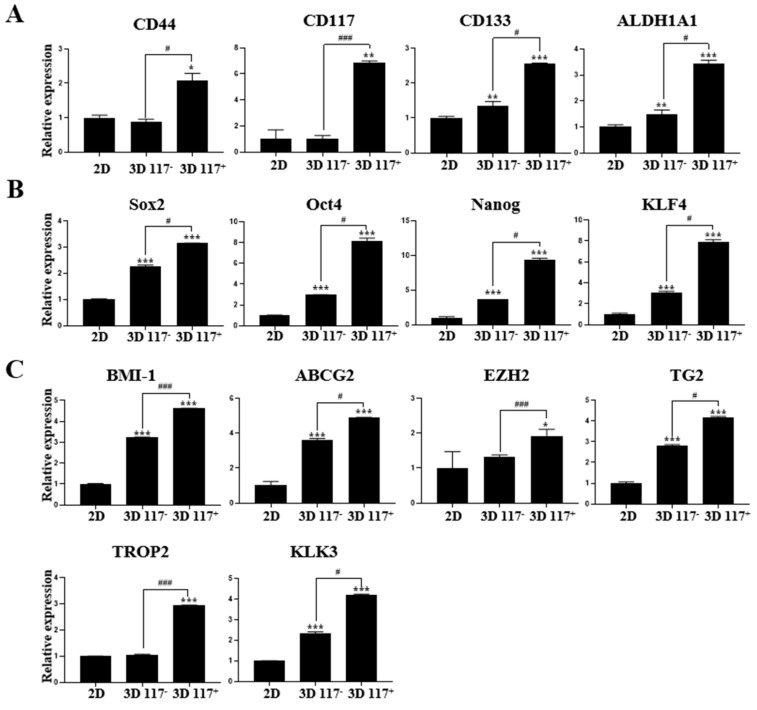
Expression of stemness- and pluripotency-related biomarkers in PC3 cell spheroids compared with those in 2D cell cultures. Real-time quantitative reverse transcription PCR (qRT-PCR) for analysis of gene expression levels of (**A**) typical prostate cancer stem cell markers (CD44, CD117, CD133, and ALDH1A1), (**B**) stemness- and pluripotency-regulating transcription factors (Sox2, Nanog, Oct4, and KLF4), and (**C**) stemness- and pluripotency-related factors (Bmi-1, ABCG2, EZH2, TG2, Trop2, and KLK3) in 2D-cultured cells as well as CD117^−^ and CD117^+^ cells from the 3D spheroids. Bar graphs show the relative gene expression in these cells. GAPDH was used as a housekeeping gene for qRT-PCR data normalization. Data represent the mean ± standard deviation of three independent experiments. * *p* < 0.05, ** *p* < 0.01, and *** *p* < 0.001 vs. 2D; ^#^ *p* < 0.05 and ^###^ *p* < 0.001 vs. CD117^−^ cells from the 3D spheroids.

First, the gene expression of typical prostate CSC surface markers, such as CD44, CD117, CD133, and aldehyde dehydrogenase 1 family member A1 (ALDH1A1), was robustly elevated in 3D cultures compared to 2D cultures, especially in isolated CD117^+^ cells (Figure 8A). Notably, in isolated CD117^+^ cells from the 3D culture, the rates of CD44, CD117, CD133, and ALDH1A1 expression were significantly increased 2.1-fold (*p* < 0.05), 6.9-fold (*p* < 0.01), 2.6-fold (*p* < 0.001), and 3.4-fold (*p* < 0.001), respectively, compared with those in 2D culture (Figure 8A). Moreover, in isolated CD117^−^ cells from the 3D culture, the rates of CD133 and ALDH1A1 expression increased 1.3-fold (*p* < 0.01) and 1.5-fold (*p* < 0.01), respectively, compared with those in the 2D culture (Figure 8A). Interestingly, in isolated CD117^+^ cells from the 3D culture, the rates of CD44, CD117, CD133, and ALDH1A1 expression increased 2.3-fold (*p* < 0.05), 6.9-fold (*p* < 0.001), 2.0-fold (*p* < 0.05), and 2.3-fold (*p* < 0.05), respectively, compared with those in the isolated CD117^−^ cells from the 3D culture (Figure 8A).

The expression of critical stemness- and pluripotency-regulating transcription factors, sex-determining region Y-box 2 (Sox2), octamer-binding transcription factor 4 (Oct4), homeobox Nanog transcription factor (Nanog), and Krüppel-like factor 4 (KLF4), was robustly augmented in 3D cultures compared to 2D cultures (Figure 8B). In isolated CD117^+^ cells from 3D culture, the rates of Sox2, Oct4, Nanog, and KLF4 expression increased 3.2-fold (*p* < 0.001), 8.1-fold (*p* < 0.001), 9.4-fold (*p* < 0.001), and 7.9-fold (*p* < 0.001), respectively, compared with those in 2D culture (Figure 8B). Notably, in isolated CD117^−^ cells from the 3D culture, the rates of Sox2, Oct4, Nanog, and KLF4 expression increased 2.2-fold (*p* < 0.001), 3.0-fold (*p* < 0.001), 3.6-fold (*p* < 0.001), and 3.1-fold (*p* < 0.001), respectively, compared with those in 2D culture (Figure 8B). In isolated CD117^+^ cells from the 3D culture, the rates of Sox2, Oct4, Nanog, and KLF4 expression increased 1.5-fold (*p* < 0.05), 2.7-fold (*p* < 0.05), 2.6-fold (*p* < 0.05), and 2.5-fold (*p* < 0.05), respectively, compared with those in the isolated CD117^−^ cells from the 3D culture (Figure 8B).

The expressions of crucial regulators of stemness and pluripotency in PC, B-cell-specific Moloney murine leukemia virus integration site 1 (Bmi-1), ATP binding cassette sub-family G member 2 (ABCG2), enhancer of zeste homolog 2 (EZH2), transglutaminase 2 (TG2), trophoblast cell-surface antigen 2 (Trop2), and kallikrein-related peptidase 3 (KLK3) were robustly augmented in 3D cultures compared with those in 2D cultures. In isolated CD117^+^ cells from the 3D cultures, the expression rates of Bmi-1, ABCG2, EZH2, TG2, Trop2, and KLK3 increased 4.6-fold (*p* < 0.001), 4.9-fold (*p* < 0.001), 1.9-fold (*p* < 0.05), 4.2-fold (*p* < 0.001), 2.9-fold (*p* < 0.001), and 4.2-fold (*p* < 0.001), respectively, compared with those in 2D cultures (Figure 8C). Notably, in isolated CD117^−^ cells from the 3D culture, the rates of Bmi-1, ABCG2, TG2, and KLK3 expression increased 3.2-fold (*p* < 0.001), 3.6-fold (*p* < 0.001), 2.8-fold (*p* < 0.001), and 2.3-fold (*p* < 0.001), respectively, compared with those in the 2D culture (Figure 8C). In isolated CD117^+^ cells from the 3D culture, the rates of Bmi-1, ABCG2, EZH2, TG2, Trop2, and KLK3 expression increased 1.4-fold (*p* < 0.001), 1.4-fold (*p* < 0.05), 1.5-fold (*p* < 0.001), 1.5-fold (*p* < 0.05), 2.9-fold (*p* < 0.001), and 1.8-fold (*p* < 0.05), respectively, compared with those in the isolated CD117^−^ cells from the 3D culture (Figure 8C).

### 2.8. Aggressiveness of PC Cells Was Reinforced in MC-B Hydrogels 

Tumor cells grown in 3D models that can efficiently reproduce the characteristics of the in vivo tissue milieu are believed to exhibit more malignant phenotypes than those grown in traditional 2D monolayer cultures. Thus, we hypothesized that multicellular 3D PC spheroids generated in MC-B hydrogels would display expedited progression compared with that displayed by 2D-cultured cells. We evaluated the expression of molecules essential for tumor aggressiveness to explore the molecular mechanism by which the 3D niche provided by the MC-B hydrogels regulates malignant cell behavior and tumor progression in PC.

**Figure 9:**
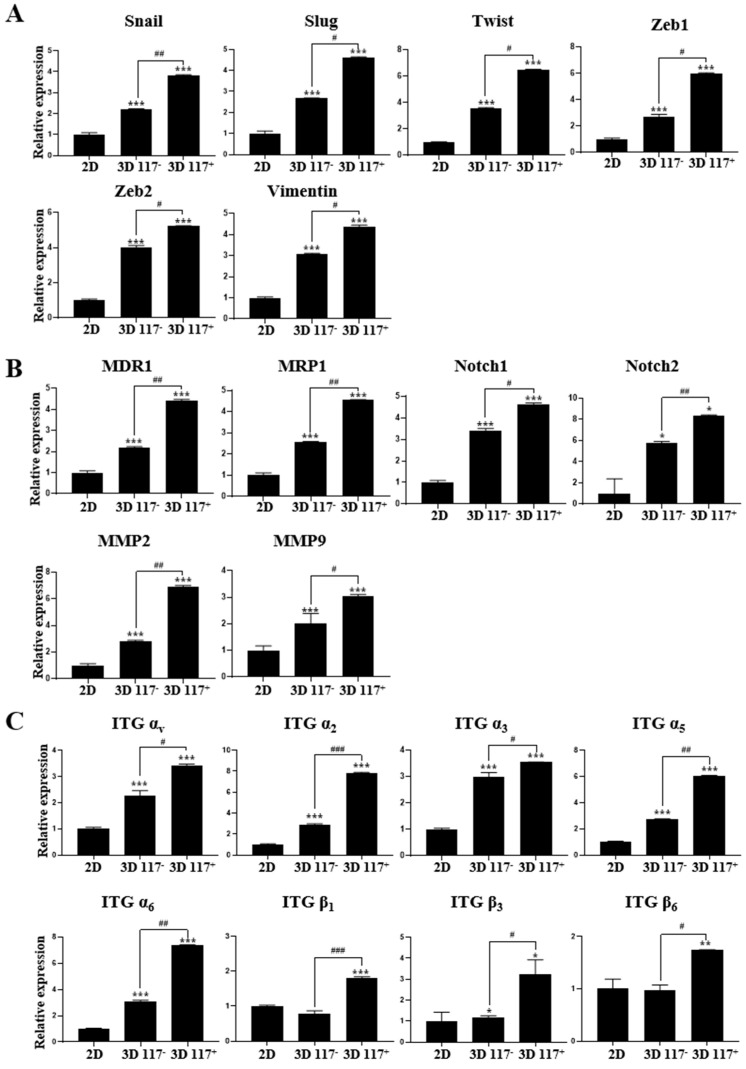
Expression of molecules associated with cancer aggressiveness in PC3 cell spheroids compared with those in 2D cell cultures. (**A**) Real-time quantitative reverse transcription PCR (qRT-PCR) gene expression analysis of epithelial–mesenchymal transition (EMT)-driving transcription factors (Snail, Slug, Twist, Zeb1, and Zeb2) and vimentin, a key biomarker of EMT, in 2D-cultured cells as well as CD117^−^ and CD117^+^ cells from 3D PC3 spheroids. (**B**) Expressions of genes associated with cancer aggressiveness (Notch1, Notch2, MMP2, MMP9, MDR1, and MRP1) by qRT-PCR analysis in 2D-cultured cells as well as CD117^−^ and CD117^+^ cells from the 3D PC3 spheroids. (**C**) Expression of multiple integrin subtypes (α_v_, α_2_, α_3_, α_5_, α_6_, β_1_, β_3_, and β_6_) by qRT-PCR analysis in 2D-cultured cells as well as CD117^−^ and CD117^+^ cells from the 3D PC3 spheroids. Bar graphs plot the densitometry quantitation of mRNA expressions normalized to GAPDH. Data represent the mean ± standard deviation of three independent experiments. * *p* < 0.05, ** *p* < 0.01, ***p < 0.001 vs. 2D; ^#^ *p* < 0.05, ^##^ *p* < 0.01, and ^###^ *p* < 0.001 vs. CD117^−^ cells from the 3D spheroids.

Multiple lines of evidence have established that epithelial–mesenchymal transition (EMT) is vital for tumor growth, progression, invasion, dissemination, metastasis, and drug resistance [29,30]. The isolated CD117^+^ cells from the 3D culture in MC-B hydrogels significantly upregulated the expression of EMT-related molecules, such as Snail, Slug, Twist, Zeb1, Zeb2, and vimentin, by 3.8-fold (*p* < 0.001), 4.6-fold (*p* < 0.001), 6.5-fold (*p* < 0.001), 6.0-fold (*p* < 0.001), 5.2-fold (*p* < 0.001), and 4.4-fold (*p* < 0.001), respectively, compared to the 2D-cultured cells (Figure 9A). Furthermore, in isolated CD117^−^ cells from the 3D cultures, the Snail, Slug, Twist, Zeb1, Zeb2, and vimentin expression levels were 2.2-fold (*p* < 0.001), 2.7-fold (*p* < 0.001), 3.5-fold (*p* < 0.001), 3.0-fold (*p* < 0.001), 4.0-fold (*p* < 0.001), and 3.1-fold (*p* < 0.001) higher, respectively, than the 2D cultured cells (Figure 9A). Interestingly, in isolated CD117^+^ cells from the 3D cultures, the Snail, Slug, Twist, Zeb1, Zeb2, and vimentin expression levels were 1.7-fold (*p* < 0.01), 1.7-fold (*p* < 0.05), 1.9-fold (*p* < 0.05), 2.2-fold (*p* < 0.05), 1.3-fold (*p* < 0.05), and 1.4-fold (*p* < 0.05) higher than those of the respective values observed in the isolated CD117^−^ cells from the 3D culture (Figure 9A).

To investigate the effects of the 3D niche provided by the MC-B hydrogels on the expression of multidrug-resistance-related genes, which play a critical role in the acquisition of chemoresistance in multiple cancers, their expression in cells grown in 2D or 3D cultures was examined. The genes examined encode multidrug resistance 1 (MDR1) and multidrug-resistance-associated protein 1 (MRP1). The expression of MDR1 and MRP1 was highly upregulated by 4.4-fold (*p* < 0.001) and 4.5-fold (*p* < 0.001), respectively, in CD117^+^ cells isolated from the 3D-cultured PC3 cells compared to the 2D-cultured controls (Figure 9B). Furthermore, in isolated CD117^−^ cells from the 3D cultures, MDR1 and MRP1 gene expression levels were 2.2-fold (*p* < 0.001) and 2.6-fold (*p* < 0.001) higher, respectively, compared to the 2D-cultured cells (Figure 9B). Interestingly, in isolated CD117^+^ cells from the 3D culture, the MDR1 and MRP1 expression levels were 2.0-fold (*p* < 0.01) and 1.7-fold (*p* < 0.01) higher than the respective values observed in the isolated CD117^−^ cells from the 3D culture (Figure 9B).

Activation of the Notch signaling pathway is important for the proliferation and progression of various tumor cell types, including PC, and for CSC maintenance [31]. Thus, we evaluated the expression of Notch1 and Notch2 to further explore the molecular mechanism by which the 3D microenvironment in the MC-B hydrogels regulates tumor aggressiveness in PC. As shown in Figure 9B, in the isolated CD117^+^ cells from the 3D culture, the levels of Notch1 and Notch2 expression increased 4.7-fold (*p* < 0.001) and 8.4-fold (*p* < 0.05), respectively, compared with those in the 2D culture (Figure 9B). Notably, in isolated CD117^−^ cells obtained from the 3D cultures, the levels of Notch1 and Notch2 expression were 3.4-fold (*p* < 0.001) and 5.8-fold (*p* < 0.05) higher, respectively, than those in 2D cultures (Figure 9B). In isolated CD117^+^ cells from the 3D cultures, the levels of Notch1 and Notch2 expression were increased 1.4-fold (*p* < 0.05) and 1.4-fold (*p* < 0.01), respectively, compared with those in the isolated CD117^−^ cells from the 3D culture (Figure 9B).

During cancer metastasis, tumor cells invade blood vessels and degrade the ECM to create a path to new distant loci. The degradation of blood vessels and ECM is mediated through the activity of matrix metalloproteinases (MMP) [32]. Hence, we evaluated the expression of MMP2 and MMP9 to further explore the molecular mechanism by which the 3D microenvironment in the MC-B hydrogels regulates tumor aggressiveness in PC. As shown in Figure 9B, in the isolated CD117^+^ cells from the 3D culture, the MMP2 and MMP9 expression levels increased 6.9-fold (*p* < 0.001) and 3.0-fold (*p* < 0.001), respectively, compared with those in the 2D culture (Figure 9B). Notably, in isolated CD117^−^ cells obtained from the 3D cultures, the MMP2 and MMP9 expression levels were 2.8-fold (*p* < 0.001) and 2.0-fold (*p* < 0.001) higher, respectively, than those in 2D cultures (Figure 9B). In isolated CD117^+^ cells from the 3D cultures, the MMP2 and MMP9 expression levels were increased 2.5-fold (*p* < 0.01) and 1.5-fold (*p* < 0.05), respectively, compared with those in the isolated CD117^−^ cells from the 3D culture (Figure 9B).

Integrins play a key role in the physiology and integrity of cells and tissues through the interaction of cells with the surrounding ECM, and contribute to cancer progression and aggressiveness [33,34]. Thus, we evaluated the expression of multiple integrin subtypes to further explore the molecular mechanism by which the 3D niche in the MC-B hydrogels regulates tumor aggressiveness in PC cells grown in 2D or 3D cultures. As shown in Figure 9C, in the isolated CD117^+^ cells from the 3D culture, integrin α_v_, α_2_, α_3_, α_5_, and α_6_ expression levels increased 3.4-fold (*p* < 0.001), 7.8-fold (*p* < 0.001), 3.6-fold (*p* < 0.001), 6.0-fold (*p* < 0.001), and 7.3-fold (*p* < 0.001), respectively, compared with those in isolated CD117^+^ cells from the 2D culture (Figure 9C). Notably, in isolated CD117^−^ cells obtained from the 3D cultures, integrin α_v_, α_2_, α_3_, α_5_, and α_6_ expression levels were 2.3-fold (*p* < 0.001), 2.9-fold (*p* < 0.001), 3.0-fold (*p* < 0.001), 2.7-fold (*p* < 0.001), and 3.1-fold (*p* < 0.001) higher, respectively, compared with those in the isolated CD117^−^ cells in the 2D culture (Figure 9C). In isolated CD117^+^ cells from the 3D cultures, integrin α_v_, α_2_, α_3_, α_5_, and α_6_ expression levels increased by 1.5-fold (*p* < 0.05), 2.7-fold (*p* < 0.001), 1.2-fold (*p* < 0.05), 2.2-fold (*p* < 0.01), and 2.4-fold (*p* < 0.01), respectively, compared with those in the isolated CD117^−^ cells from the 3D culture (Figure 9C). Furthermore, in the isolated CD117^+^ cells from the 3D culture, integrin β_1_, β_3_, and β_6_ expression levels increased 1.8-fold (*p* < 0.001), 3.2-fold (*p* < 0.05), and 1.7-fold (*p* < 0.01), respectively, compared with those in the 2D culture (Figure 9C). Notably, in isolated CD117^−^ cells obtained from the 3D cultures, integrin β_3_ expression level increased 1.2-fold (*p* < 0.05) compared with those in the 2D culture (Figure 9C). In isolated CD117^+^ cells from the 3D cultures, integrin β_1_, β_3_, and β_6_ expression increased 2.3-fold (*p* < 0.001), 2.7-fold (*p* < 0.05), and 1.7-fold (*p* < 0.05), respectively, compared with those in the isolated CD117^−^ cells from the 3D culture (Figure 9C).

## 3. Discussion 

PC is one of the most common cancers in men worldwide and accounts for a large proportion of all cancer-related deaths [35]. The high failure rate of PC drug development is attributable to the lack of reliable preclinical in vitro testing models. These models should closely mirror the disease, thereby considerably improving the likelihood of success of future clinical trials. In this study, we developed a robust 3D PC cell culture technology for simple, rapid, and inexpensive ex vivo drug sensitivity testing and for enriching CSCs. In this study, we demonstrated that a 3D PC cell culture system using MC-B hydrogels displays several characteristics. These characteristics include the following: increased cell proliferation, migration, invasion, colony formation, and chemoresistance; higher expression of multidrug-resistance- and cancer-stemness-related genes; elevated levels of molecules linked to tumor progression (Snail, Slug, Twist, Zeb1, Zeb2, Notch1, Notch2, MMP2, MMP9, Sox2, Oct4, Nanog, KLF4, and integrins); and substantial enrichment of prostate CSCs compared to traditional 2D cultures. Thus, our results highlight the potential benefits of MC-B hydrogels in the development of strategies for new anti-cancer drug discovery and targeted CSC therapy for PC.

The tumor microenvironment comprises a complex tissue matrix that includes various components, such as the ECM, and a signaling network composed of cytokines, chemokines, growth factors, hormones, cell adhesion molecules, and matrix metalloproteinases. These elements are critical for regulating cell-to-cell communication and interactions between cells and the ECM. The tumor microenvironment substantially influences tumor progression by supporting cellular processes, such as survival, adhesion, proliferation, differentiation, migration, invasion, metastasis, tumor recurrence, and the development of chemoradiotherapy resistance, underscoring the complexity of cancer biology and highlighting potential therapeutic targets within the microenvironment [36,37]. Three-dimensional cell culture systems more accurately mimic critical biological phenomena observed in vivo, such as gene and protein expression, as well as cellular processes, including survival, proliferation, adhesion, migration, development, and differentiation [38,39]. Thus, these systems offer a nuanced representation of both functional and morphological aspects of tissues, enhancing the relevance of laboratory studies in real-world biological contexts. In this context, 3D cell culture has rapidly become the preferred platform for creating in vitro tumor models that closely simulate the behavior of tumor cells in vivo. This platform offers a viable alternative to traditional 2D cell cultures, which are often inadequate because of their limited ability to replicate the complex 3D architecture of tumor tissues. 

Researchers have extensively explored novel anti-cancer treatments, including drug screening, primarily in animal models [40,41,42]. However, high costs, lengthy and complex processes, discrepancies in clinical outcomes owing to many factors, such as compromised immune systems that are commonly observed in animal models, and ethical concerns, such as discomfort or pain in animals under certain experimental conditions, limit the effectiveness and applicability of these animal-based studies [43]. In particular, the average rate of concordance between animal models and clinical trials is barely 8% [44,45]. Furthermore, 2D monolayer cell cultures, despite their widespread use in anti-cancer drug screening, often produce many false-positive or false-negative test results because they do not accurately mimic the complex in vivo environment, including the lack of cell-to-cell and cell-to-matrix interactions and apicobasal polarity. Therefore, switching from 2D cultures and animal models to 3D in vitro cell culture is emerging as a crucial technique in cancer research, thereby enhancing the understanding of the biological mechanisms underlying cancer progression, such as drug resistance and metastasis, as well as facilitating the identification of biomarkers and development of therapeutic strategies [46]. Despite extensive research on 3D in vitro PC models to replicate cancer complexity [47,48,49,50,51], no definitive and ideal model has yet been established, and ongoing detailed studies continue to reveal new and intriguing information. 

Multidrug resistance is a considerable hurdle in cancer treatment, characterized by cancer cells gaining the ability to withstand various anti-cancer drugs. This phenomenon is the key factor for relapse and mortality among cancer patients [52]. Resistance to chemotherapy involves several mechanisms, including enhanced drug efflux from cells [53]. This process is facilitated by membrane transporter proteins such as MDR1, also termed permeability glycoprotein (P-glycoprotein, P-gp) or ATP-binding cassette subfamily B member 1, and MRP1, also known as ATP-binding cassette subfamily C member 1. These proteins actively pump anti-cancer drugs out of the cells, reducing their efficacy and resulting in decreased exposure to chemotherapeutic agents [54]. 

Corroborating these facts, we noted an increase in chemoresistance to anti-cancer agents in PC cells, concomitant with the upregulation of the major drug resistance genes MDR1 and MRP1 in 3D MC-B hydrogels. These observations underscore the efficacy of our PC model in replicating the tumor microenvironment more accurately than the 2D model replicates. Moreover, by day 10, the average spheroid sizes of LNCaP, DU-145, and PC3 cells exceeded 100 µm. Spheroids larger than approximately 100 μm are known to harbor an internal hypoxic zone owing to restricted oxygen, nutrient, and metabolite distribution, leading to the formation of a necrotic core [53,54]. This hypoxic tumor microenvironment is pivotal in mediating drug resistance via specific cellular signaling pathways and influences the resistance of CSCs to chemotherapy and radiotherapy [55]. These findings highlight the suitability of our MC-B hydrogel for cultivating PC cell spheroids, which could be instrumental in devising diagnostic and therapeutic approaches for PC treatment. 

In addition to the expression of typical prostate CSC biomarkers (CD44, CD117, CD133, and ALDH1A1) and crucial stemness- and pluripotency-regulating transcription factors (Sox2, Oct4, Nanog, and KLF4), the augmented expression of essential stemness- and pluripotency-associated factors in PC (Bmi-1, ABCG2, EZH2, TG2, Trop2, and KLK3) in CD117^+^ cells isolated from PC spheroids demonstrated the CSC-enrichment efficiency of our 3D PC cell culture model. Bmi-1, a polycomb-group transcriptional repressor, plays a pivotal role in numerous cellular functions, such as stem cell self-renewal and proliferation of cancer cells [56]. Frequent Bmi-1 upregulation in PC underscores its critical involvement in prostate CSC renewal and progression to malignancy [56]. ABCG2 transporters, belonging to the ATP-binding cassette (ABC) superfamily, employ ATP hydrolysis to shuttle various substrates, including chemotherapeutic drugs, steroids, and xenobiotics, from the intracellular to the extracellular environment. Elevated ABCG2 levels enhance stemness, whereas its depletion prompts lineage commitment in retinal, hematopoietic, and cardiac-side population cells [57]. Notably, ABCG2, which is recognized as a marker of prostate CSCs, plays a crucial role in maintaining prostate stem cell populations [57]. EZH2 functions as a catalytic enzyme and plays an important role in histone methylation. ABCG2 directly methylates promoters of key stemness- and pluripotency-regulating transcription factors, such as Oct4, Sox2, and Nanog, thereby maintaining stem cell pluripotency [58,59]. TG2 is a critical factor for cancer cell survival, exhibiting heightened levels within cancer cells and further concentrated within cancer stem cells to sustain the cancer stem cell phenotype [60,61]. TG2 levels are elevated in advanced PC and are associated with anti-androgen resistance in prostate CSCs [62,63]. Trop2 is associated with prostate CSCs in both mice and humans. Cells expressing Trop2 demonstrate elevated levels of the stem cell regulator Sox2 and manifest stem cell-like characteristics, such as self-renewal and tissue regeneration, within the prostate [64,65,66,67]. KLK3, also known as a PSA, is a single-chain glycoprotein and protease produced by prostate epithelial cells [68]. KLK3 is the most extensively studied serum biomarker used for early prostate cancer screening, clinical staging, and therapeutic response monitoring. PSA markers are strongly linked to the capacity of prostate cancer cells to self-renew and express stemness genes [63]. Overall, these results demonstrate that our MC-B hydrogel is well suited for cultivating PC cell spheroids, providing an effective method for inducing and enriching prostate CSCs. 

Although the existence of CSCs is debatable, evidence suggests that they contribute substantially to chemoradioresistance and treatment failure [69]. CSCs are implicated in acquired drug resistance after chemotherapy and serve as tumor-initiating cells during recurrence and metastasis, owing to their DNA repair capacity, multidrug resistance, and self-renewal ability [70,71]. Targeting CSCs is crucial for preventing cancer relapse. However, current therapies lack specific biomarkers for effective targeting [72]. Thus, the development of innovative CSC-targeting methods may be an effective strategy for the treatment of multidrug-resistant, metastatic, and recurrent PC [72]. Hence, a pivotal approach involves the development of an efficient and cost-effective method for CSC enrichment. 

Over the last decade, several strategies using conventional 2D cell culture platforms have been explored for CSC enrichment, including hypoxic culture, chemoradiotherapy stimulation, and molecule-mediated triggering [73,74]. However, 2D culture conditions limit the expansion and long-term maintenance of the clonal and differentiation capacities. They also fail to replicate the intricate CSC niche and dynamic 3D microenvironments crucial for regulating CSC fate in vivo [75].

Although notable strides have been made in conceiving and designing CSC enrichment methods, there is a surge of interest in 3D cell culture as a means of enhancing the efficacy of CSC enrichment, aiming to address the limitations of 2D-based approaches. Various strategies, categorized as non-scaffold-based or scaffold-based techniques, within the realm of 3D cell culture using diverse scaffold materials, such as hydrogels and nanofibrous scaffolds, have demonstrated superior benefits in enriching and characterizing CSCs in vitro compared with those of traditional 2D culture methods [76,77,78,79,80]. In recent decades, consistent with our findings, 3D cell culture techniques have been employed as effective strategies for enriching and culturing prostate CSCs to advance research and therapy development [48,81]. Despite these advancements, refining techniques to precisely enrich the desired prostate CSC population remains a significant challenge.

Cancer metastasis, the spread of a tumor to a distant site, is a hallmark of cancer and the primary cause of cancer morbidity and mortality, accounting for approximately 90% of cancer-related deaths [81]. EMT, a characteristic of most metastatic cancers, is a complex process that transforms epithelial cells into mesenchymal cells, leading to cancer cell migration and invasion. EMT is linked to metastasis and cancer stem cell dynamics and consequently fuels tumor invasion, heterogeneity, and chemoresistance [29,30]. In this study, we showed that cells cultured in MC-B hydrogels, particularly isolated CD117^+^ CSCs, strikingly upregulated the expression of pivotal EMT transcription factors such as Snail, Slug, Twist, Zeb1, and Zeb2, as well as vimentin, a key biomarker of EMT. The Notch pathway is one of the most extensively explored therapeutic targets for tumor cells given its crucial role in cell proliferation, aggressiveness, chemoresistance, and stem cell propagation across various primary and metastatic tumors. The molecular mechanisms underlying Notch-signaling-induced chemoresistance involve the induction of EMT, tumor stem cell formation, and heightened expression of MDR proteins such as MDR1 and MRP1 [31,82,83,84]. MMPs play pivotal roles in various physiological and pathological processes, including morphogenesis, wound healing, inflammation, cancer invasion, and metastasis [85]. Structurally, MMPs are divided into several subtypes, with MMP2 and MMP9 belonging to the gelatinase family, which primarily degrade gelatin and collagen IV and V in the ECM and basement membrane (BM), respectively, through their proteolytic functions [86]. In cancer, the overproduction or increased activity of MMP2/9 leads to the degradation of ECM and BM, thereby facilitating tumor cell invasion and metastasis to distant organs [87]. Additionally, MMP2/9 have been implicated in cancer development and progression through their roles in apoptosis, proliferation, and angiogenesis [88,89]

Consequently, numerous studies have focused on devising targeted therapeutic approaches aimed at inhibiting the Notch pathway, EMT-inducing transcription factors, and molecules associated with cancer stemness to enhance the efficacy of cancer treatment. Given the prominent role of these molecules in driving the EMT, our results are significant because we demonstrated that MC-B hydrogels serve as an adequate scaffold for cultivating PC cell spheroids and provide a favorable niche for inducing the EMT.

The ECM is a crucial component of the tumor microenvironment and is composed of extracellular macromolecules and minerals. Initially viewed merely as a structural scaffold, the influence of the ECM on diverse cellular processes, particularly cancer, is now recognized [90]. Integrins, acting as transmembrane receptors for ECM proteins, orchestrate crucial cellular processes, such as survival, proliferation, migration, gene expression, and activation of growth factor receptors [91]. In PC, tumor cells exhibit an aberrant integrin profile and reside within a distinctly altered ECM environment. These alterations have significant implications, given the capacity of each integrin to modulate specific cellular functions [91]. Integrin plays a more crucial role in the invasion of PC3 cells than in other cells [92,93,94]. Integrins have emerged as promising targets for PC biomarkers and therapeutics because they are involved in regulating cell adhesion and migration in PC and facilitate intracellular trafficking, leading to cell proliferation, invasion, tumor growth, neo-angiogenesis, and metastasis [91,94].

The α_v_β_3_ integrin, involved in cell adhesion, migration, and invasion by binding to ECM proteins, such as vitronectin and fibronectin, has been shown to promote PC cell migration and invasion as well as angiogenesis, suggesting that its expression is associated with increased metastatic potential [91,94]. The integrin also contributes to the survival and proliferation of prostate cancer cells by activating intracellular signaling pathways such as the PI3K/Akt pathway [94]. The α_2_β_1_ integrin, a recognized receptor for collagen, is prominently expressed in PC and its activation/phosphorylation has been associated with PC progression [91]. Integrin α_2_β_1_ regulates self-renewal in prostate CSCs by interacting with ECM proteins, particularly collagen, to maintain the stem cell niche and support CSC self-renewal, facilitating tumor initiation by CSCs [91]. Integrin α_3_β_1_ interaction with laminin activates signaling pathways, promotes CSC proliferation, and drives tumor growth and progression, thereby facilitating effective interaction of prostate CSCs with the tumor microenvironment, which is crucial for maintaining the stem cell niche and ensuring CSC maintenance and protection [95]. Integrin α_5_β_1_ binds primarily to fibronectin, which is crucial for cell adhesion, migration, and survival. Elevated α_5_β_1_ levels in PC tissues correlate with heightened tumor aggressiveness and enhanced signaling that promotes proliferation, survival, increased migration, and resistance to apoptosis, thereby facilitating tumor progression and metastasis [96]. Integrin α_6_β_1_ binds to laminin and subsequently enhances cell adhesion and migration. The integrin also influences PC cell responses to growth factors and promotes tumor growth [91]. Integrin β_1_ is crucial for PC cell adhesion, survival, and apoptosis resistance, mediating signals between the ECM and intracellular pathways [97]. Integrin β_3_ promotes PC metastasis by facilitating cancer cell adhesion to the ECM of distant organs, aiding invasion and colonization at secondary sites [98]. Integrin β_6_ forms α_v_β_6_ heterodimers, known for specific epithelial tissue expression and roles in cancer biology [99]. Therefore, the development of novel therapeutic agents that target the functions of these integrins holds promise for the inhibition of PC growth and metastasis. Collectively, our results on integrin expression demonstrate that MC-B hydrogels offer an optimal niche for integrin expression in PC cells, serving as an effective in vitro model for mimicking PC progression associated with integrin activation.

Interestingly, we observed that our MC-B hydrogels provided a favorable environment for several key aspects: (1) survival, proliferation, colony formation, migration, invasion, and CSC formation of PC cells; (2) significant enrichment of prostate CSCs; and (3) acquisition of an enhanced malignant phenotype in PC cells, including chemoresistance, metastatic potential, and stemness, which can be leveraged for developing diagnostic, treatment, and preventive strategies against PC. In our study, we introduced a novel, rapid, and efficient approach for enriching prostate CSCs while simultaneously engineering a 3D in vitro model of PC chemoresistance and aggressiveness, with a particular focus on prostate CSCs. This 3D model mimics the in vivo microenvironment more accurately than the traditional monolayer models. Therefore, our biomimetic MC-B hydrogel matrix offers innovative insights into the role of prostate CSCs in metastasis, chemotherapeutic resistance, and recurrence, thus overcoming the limitations of 2D cell cultures. Future research should focus on refining the CSC enrichment techniques to enhance our understanding of prostate CSC biology. These advances hold promise for the development of CSC-targeted therapies for PC, potentially leading to improved clinical outcomes.

## 4. Materials and Methods

### 4.1. Cell Culture

Human PC cell lines (LNCaP, DU-145, and PC3) were obtained from the Korean Cell Line Bank (KCLB, Seoul, Republic of Korea). All cell lines were cultured and maintained in RPMI 1640 (Hyclone, Chicago, IL, USA) supplemented with 10% fetal bovine serum (FBS, Welgene, Daegu, Republic of Korea) and 1% penicillin-streptomycin (Gibco/Thermo Fisher Scientific, Carlsbad, CA, USA) in a 5% CO_2_ humidified atmosphere at 37 °C. Sub-confluent cells were harvested using trypsin-EDTA (Welgene) and used for further experiments. The medium was replaced every three days.

### 4.2. Synthesis of Hydrogels for 3D Cell Culture

MC-B hydrogels for 3D cell culture were prepared as previously described [100,101]. Briefly, 50 mg/mL sodium alginate was dissolved in deionized water for 16 h at room temperature (RT) to prepare a 5% alginate stock solution, which was autoclaved before use. Agarose (Affymetrix, Cleveland, OH, USA) was added to distilled water and heated to 100 °C to achieve a 2% agarose stock solution. Fish collagen peptide (Geltech, Busan, Republic of Korea) was added to distilled water at 0.3 g/mL, vortexed to dissolve, and resulted in a 30% marine collagen stock solution. A 565-μL volume of cells resuspended in culture medium (1 × 10^5^ cells/mL) was mixed with 240 μL of 5% sodium alginate solution in a 1.5-mL microcentrifuge tube at RT. This solution was then combined with 320 μL of 30% MC stock solution at RT to obtain 8% MC/1% alginate-solution-containing cells. These cell suspensions were then blended carefully with 75 μL of 2% agarose solution at 35–40 °C to avoid cell damage. For the gelation of hydrogel solutions containing cells, the solutions were vortexed briefly, pipetted into 1-mL syringes, and ultimately incubated at 4 °C for 5–10 min. The gelled hydrogels were then transferred to the wells of 24-well plates (SPL Life Sciences, Pocheon, Republic of Korea) containing 1.5 mL of RPMI 1640 (Hyclone) and incubated at 37 °C. The medium was changed every two days.

### 4.3. Spheroid Growth Assay 

To evaluate the effects of the MC-B hydrogels on the formation and growth of multicellular spheroids, LNCaP, DU-145, and PC3 cells were cultured for 3, 5, 7, 10, and 14 days. The sizes of the spheroids were measured at the desired time points using a phase-contrast microscope (IX70, Olympus, Tokyo, Japan). At least 20 spheroids on each hydrogel were photographed and their diameters were measured. The diameter of a spheroid was defined as the average length of the diameters measured at two-degree intervals joining two outline points and passing them through the centroid [101]. The spheroid diameter was quantified and analyzed using image analysis software (ImageJ, version 1.52a, National Institute of Health, Bethesda, MD, USA). 

### 4.4. Cell Proliferation Assay

Cell proliferation assays of 2D and 3D cell cultures were performed as previously described [101]. Briefly, for the 2D culture, LNCaP, DU-145, and PC3 cells were seeded at a density of approximately 1 × 10^4^ cells/well into 96-well plates (SPL Life Sciences), and for the 3D culture, the cells were seeded at a density of 1 × 10^5^ cells/mL. These cells were cultured in a complete medium containing 10% FBS (Welgene) for 1, 3, 5, 7, 10, and 14 days. To measure cell proliferation, the WST-1 colorimetric assay was performed as per the manufacturer’s instructions (Daeil Lab Service, Seoul, Republic of Korea) in 96-well plates (SPL Life Sciences). Briefly, the plates were washed with phosphate-buffered saline (PBS). Ten microliters of WST-1 reagent were added to each well, and the plates were incubated for 1 h in a humidified chamber at 37 °C in 5% CO_2_. To quantify the metabolic cells, formazan absorbance was measured at 450 nm using a microplate reader (Tecan, Männedorf, Switzerland). The cell viability was calculated as a percentage of the 2D control cell population. Cell morphology and spheroid size were assessed at the desired time points using a phase-contrast microscope (IX70; Olympus). All experiments were performed independently at least three times.

### 4.5. Colony Forming Assay

LNCaP and PC3 cells that were 2D- and 3D-cultured for seven days were seeded into six-well plates (SPL Life Sciences) at a density of 200 cells/well as described previously [101]. They were grown for seven days, at which time suitably sized colonies were usually observed. Colonies were fixed with 100% methanol for 20 min at −20 °C and washed with PBS. The colonies were subsequently stained with 0.5% crystal violet solution (Sigma-Aldrich, St Louis, MO, USA) for 5 min. After a second wash with PBS, the plates were allowed to dry overnight. Stained colonies were counted to determine the number of colony-forming units. Each experiment was repeated thrice.

### 4.6. Wound Healing Assay

A wound healing assay was performed as previously described [101]. LNCaP, DU-145, and PC3 cells cultured in the MC-B hydrogel for seven days were seeded at a density of 5 × 10^5^ cells/well in six-well plates (SPL Life Sciences), and 2D-cultured cells were used as controls. When 2D- and 3D-cultured cells attained complete confluence, the medium was changed to a starvation medium containing only 1% FBS (Welgene). After scratching the wounds in each well with a scratcher (SPL Life Sciences), the wells were rinsed with PBS to remove cellular debris and avoid the re-establishment of displaced cells. The scratch closure was monitored and imaged at the desired time points using a phase-contrast microscope (IX70, Olympus). Each experiment was repeated thrice. 

### 4.7. Hydrogel Invasion Assay

The hydrogel invasion assay was performed as previously described [101]. LNCaP, DU-145, and PC3 cells cultured in MC-B hydrogels for seven days at 37 °C in a 5% CO_2_ humidified incubator were seeded at a density of 5 × 10^5^ cells/well on the surface of freshly prepared MC-B hydrogels in six-well plates (SPL Life Sciences). The 2D-cultured cells were used as controls. For confocal laser scanning microscopy, hydrogels containing 2D- and 3D-cultured cells were washed with PBS and then fixed for 20 min with cold 4% paraformaldehyde (PFA, Sigma-Aldrich) in PBS. The fixative was removed by washing the hydrogels three times for 5 min each with cold PBS, followed by permeabilization with 0.1% Triton X-100 (Sigma-Aldrich) in PBS for 5 min. After washing with cold PBS, the samples were blocked with 2% bovine serum albumin (BSA; Sigma-Aldrich) for 1 h at RT. Excess solution was removed, and hydrogels were incubated for 1 h at RT with 1:150 diluted FITC-phalloidin (Promega, Madison, WI, USA), rinsed in cold PBS, and mounted on glass slides using Vectashield^®^ containing 4′,6-diamidino-2-phenylindole (DAPI; Vector Laboratories, Burlingame, CA, USA). The cell fluorescence was observed using a confocal laser scanning microscope (LSM900; Carl Zeiss, Jena, Germany). To examine the depth of cell invasion, a stack of confocal images was collected using a step size of 5 μm. Z-stack images were used to generate reconstructed 3D projection images.

### 4.8. Evaluation of Immunofluorescence Using Confocal Microscopy 

LNCaP and PC3 cells were cultured in MC-B hydrogels for seven days at 37 °C in a 5% CO_2_ humidified incubator. The cells were then washed twice with PBS, fixed with cold 4% PFA (Sigma-Aldrich) in PBS for 20 min, permeabilized with 0.1% Triton X-100 (Sigma-Aldrich) in PBS for 10 min, and blocked with 2% BSA (Sigma-Aldrich) for 1 h at RT. Primary antibody CD44 (1:200; BioLegend, San Diego, CA, USA) was added and incubated at 4 °C overnight. Goat anti-rat secondary antibodies labeled with FSD-647 (1:100; BioActs, Republic of Korea) were added and incubated for 1 h. The nucleus was stained with DAPI (Vector Laboratories). For the evaluation of 3D morphology, LNCaP, DU-145, and PC3 cells were 2D- or 3D-cultured at 37 °C in a 5% CO_2_ humidified incubator for 3, 5, and 7 days. The cells were then incubated with 1:150 diluted FITC-phalloidin (Promega) for 2 h at RT. Immunofluorescence staining was performed using a laser confocal microscope (LSM900).

### 4.9. RNA Isolation and cDNA Synthesis 

Total RNA from PC3 cells that were subjected to 2D or 3D culture for seven days was isolated using TRIzol reagent (Favorgen Biotech Corp., Pingtung, Taiwan). RNA quality and quantity were determined using a NanoDrop 2000 spectrophotometer (Thermo Scientific, Waltham, MA, USA). First-strand cDNA was synthesized with 1 µg total RNA from each sample using a HiSenScript™ RH (−) RTase cDNA Synthesis Kit (iNtRON Biotechnology, Sungnam, Republic of Korea). The reaction mixture was incubated at 45 °C for 60 min and then heated to 85 °C for 10 min to stop the reaction. 

### 4.10. Quantitative Real-Time PCR (qRT-PCR)

The Bio-Rad CFX Connect Real-Time system (Hercules, CA, USA) and the DNA-binding dye SYBR Green I with SsoAdvanced Universal SYBR Green Supermix (Bio-Rad) were used for qRT-PCR. Primers used for qRT-PCR are listed in Table 1. All samples were amplified in triplicates. Gene expression levels were calculated using the 2^−ΔΔCt^ method and normalized to GAPDH expression levels. The expression of the control sample was set to 1, and the relative expression of the other samples was calculated accordingly.

### 4.11. Flow Cytometry

To detect CSC populations, LNCaP and PC3 cells were cultured in 2D and 3D media for seven days and harvested by pipetting. They were washed with Hanks’ balanced salt solution (HBSS; Gibco/Thermo Fisher Scientific) containing 0.1% BSA (Sigma-Aldrich) and 0.1% sodium azide (Sigma-Aldrich) and filtered through a 100-μm cell strainer (SPL Life Sciences). Phenotypic analysis of cell-surface marker expression was performed using flow cytometry. Briefly, the cells were washed twice with HBSS (Gibco/Thermo Fisher Scientific) and resuspended in cell-staining buffer. Cells were immunostained for cell surface markers by incubating for 30 min with FITC-labeled anti-CD44 (1:10, Invitrogen, Life Technologies, Carlsbad, CA, USA), PE-labeled anti-CD117 (1:10; BioLegend), and APC-labeled anti-CD133 (1:10; BioLegend) monoclonal antibodies. Two-dimensional cultured cells were used as controls. Flow cytometric analysis was performed using a FACSCanto-II flow cytometer (BD Biosciences, San Jose, CA, USA). Flow cytometry data were analyzed using the FlowJo 10.3.0 (Tree Star, Ashland, OR, USA).

### 4.12. Cell Sorting

To disperse the cells in a single-cell suspension from the hydrogel matrix, 10-day MC-B hydrogels with embedded cells were thoroughly mixed with RPMI-1640 medium (Hyclone) using a pipette. The cell-polymer suspension was washed with the culture medium and filtered through a 70 μm cell strainer (SPL Life Sciences) to eliminate the hydrogel debris and all remaining cell clumps. After washing the cells with PBS at least twice, they were resuspended at a concentration of 4–5 million cells/mL. The cells were incubated with PE-labeled anti-CD117 (BioLegend) while agitating in an orbital shaker at 200 rpm for 45 min at 4 °C. The cells were then sorted through a 100 μm nozzle at a sheath pressure of 20 psi and a drop drive frequency of 30 kHz into CD117^+^ and CD117^−^ cell populations on the FACSAria III cell sorter (BD Biosciences) using FACSDiva software. A highly pure sorting modality (four-way purity sorting for FACSAria III) was selected. The flow rate during sorting was approximately 8000 events/sec. Appropriate forward and side scatter gating were used to isolate viable cells. Gates were set with reference to the negative controls. The sorting speed was adjusted to ensure a sorting efficiency of above 90%. Sorted cells were collected in 5 mL polypropylene tubes (SPL Life Sciences) containing 1 mL of collection medium (RPMI 1640 medium supplemented with 20% FBS). 

### 4.13. Chemotherapeutic Sensitivity Assay

After LNCaP and PC3 cells were 2D- and 3D-cultured for seven days, the cell culture media were replaced with serum-free media containing abiraterone (0–100 μM; Sigma-Aldrich), cisplatin (0–200 μM; Sigma-Aldrich), curcumin (0–100 μM; Sigma-Aldrich), docetaxel (0–500 nM; Sigma-Aldrich), and enzalutamide (0–100 μM; Sigma-Aldrich) for 24 and 48 h. To determine cell viability, a WST-1 assay was performed as previously described. All experiments were performed in triplicates and repeated thrice.

### 4.14. Statistical Analysis

All quantitative results are expressed as mean ± standard deviation (SD) of at least three independent experiments. Comparisons between the two groups were analyzed using Student’s *t*-test. A value of *p* < 0.05 was considered statistically significant. 

## 5. Conclusions

Herein, we present an effective 3D in vitro culture method based on an MC-B hydrogel matrix, which was developed and optimized for the growth of multicellular PC spheres derived from different PC cell lines. We demonstrated the model’s usefulness for isolating and enriching prostate CSCs. The 3D in vitro PC cell culture using the MC-B hydrogel scaffold offers several advantages, including simplicity, reproducibility, bioactivity, efficiency, and low cost. PC cells grown in the 3D culture system exhibited biochemical and physiological features: (1) enhanced cell proliferation, migration and invasion, and colony formation; (2) augmented chemotherapy resistance and upregulated expression of multidrug-resistance-related genes (MDR1 and MRP1); (3) enrichment of a prostate CSC population, evidenced by heightened expression of cancer stemness and pluripotency-related biomarkers (CD44, CD117, CD133, ALDH1A1, Sox2, Oct4, Nanog, KLF4, Bmi-1, ABCG2, EZH2, TG2, Trop2, and KLK3); (4) heightened levels of key molecules associated with tumor progression and malignancy, including EMT transcription factors (Snail, Slug, and Twist, Zeb1, and Zeb2), Notch (Notch1 and 2), and MMPs (MMP2 and MMP9); and (5) elevated expression of integrins (α_v_, α_2_, α_3_, α_5_, α_6_, β_1_, β_3_, and β_6_). Subsequent extension of our in vitro observations to an in vivo xenograft tumor model is required to determine whether 3D-culture-derived CD117^+^ PC cells would stimulate tumor growth. Furthermore, deeper investigations to fully elucidate which signaling pathways impact the growth of CD117^+^ stem-cell-like PC cells and how these pathways function at a therapeutic level can shed light on the development of novel targeted therapies. The 3D in vitro PC model is a promising in vitro research platform to study PC and prostate CSC biology and screen new anti-PC and anti-prostate CSC-targeted therapeutics. 

## Figures and Tables

**Table 1:** qRT-PCR primer names and their sequences.

Gene Name	Forward (5’-3’)	Reverse (5’-3’)
*ABCG2*	GTTCTCAGCAGCTCTTCGGCTT	TCCTCCAGACACACCACGGATA
*ALDH1*	CGGGAAAAGCAATCTGAAGAGGG	GATGCGGCTATACAACACTGGC
*BMI-1*	GGTACTTCATTGATGCCACAACC	CTGGTCTTGTGAACTTGGACATC
*CD44*	CTGCCGCTTTGCAGGTGTA	CATTGTGGGCAAGGTGCTATT
*CD117*	CACCGAAGGAGGCACTTACACA	TGCCATTCACGAGCCTGTCGTA
*CD133*	CACTACCAAGGACAAGGCGTTC	CAACGCCTCTTTGGTCTCCTTG
*EZH2*	GACCTCTGTCTTACTTGTGGAGC	CGTCAGATGGTGCCAGCAATAG
*ITGα_2_*	GTTAGCGCTCAGTCAAGGCA	GCCAAACTGTTCACTTGAAGGAC
*ITGα_3_*	AGCGCTACCTGCTCCTGGCT	GGGCAGTGAGTGGGCACAGG
*ITGα_5_*	GGGTGGTGCTGTCTACCTC	GTGGAGCGCATGCCAAGATG
*ITGα_6_*	CGAAACCAAGGTTCTGAGCCCA	CTTGGATCTCCACTGAGGCAGT
*ITGα_v_*	AGGCACCCTCCTTCTGATCC	CTTGGCATAATCTCTATTGCCTGT
*ITGβ_1_*	GCCTTACATTAGCACAACACC	CATCTCCAGCAAAGTGAAACC
*ITGβ_3_*	CTGCCGTGACGAGATTGAGT	CCTTGGGACACTCTGGCTCT
*ITGβ_6_*	TCTCCTGCGTGAGACACAAAGG	GAGCACTCCATCTTCAGAGACG
*KLF4*	CATCTCAAGGCACACCTGCGAA	TCGGTCGCATTTTTGGCACTGG
*KLK3/PSA*	CGCAAGTTCACCCTCAGAAGGT	GACGTGATACCTTGAAGCACACC
*MDR1*	GCTGTCAAGGAAGCCAATGCCT	TGCAATGGCGATCCTCTGCTTC
*MMP2*	TGACGGTAAGGACGGACTC	ATACTTCACACGGACCACTTG
*MMP9*	CAGAGATGCGTGGAGAGT	TCTTCCGAGTAGTTTTGG
*MRP1*	CCGTGTACTCCAACGCTGACAT	ATGCTGTGCGTGACCAAGATCC
*Nanog*	CTCCAACATCCTGAACCTCAGC	CGTCACACCATTGCTATTCTTCG
*Notch1*	GGTGAACTGCTCTGAGGAGATC	GGATTGCAGTCGTCCACGTTGA
*Notch2*	GTGCCTATGTCCATCTGGATGG	AGACACCTGAGTGCTGGCACAA
*Oct4*	CTTGAATCCCGAATGGAAAGGG	GTGTATATCCCAGGGTGATCCTC
*Snail*	ACTGCAACAAGGAATACCTCAG	GCACTGGTACTTCTTGACATCTG
*Slug*	TGTGACAAGGAATATGTGAGCC	TGAGCCCTCAGATTTGACCTG
*Sox2*	GCTACAGCATGATGCAGGACCA	TCTGCGAGCTGGTCATGGAGTT
*TG2*	TGTGGCACCAAGTACCTGCTCA	GCACCTTGATGAGGTTGGACTC
*Trop2*	GGACATCAAGGGCGAGTCTCTA	AGGCGCTTCATGGAGAACTTCG
*Twist*	GTCCGCAGTCTTACGAGGAG	GCTTGAGGGTCTGAATCTTGCT
*Zeb1*	TTACACCTTTGCATACAGAACCC	TTTACGATTACACCCAGACTGC
*Zeb2*	GCGATGGTCATGCAGTCAG	CAGGTGGCAGGTCATTTTCTT
*GAPDH*	GGAGAAGGCTGGGGCTCAT	TGATGGCATGGACTGTGGTC

## Data Availability

The original data presented in the study are included in the article; further inquiries can be directed to the corresponding author.
